# Metallic foreign body deep in the prevertebral space after an endomyocardial biopsy: a case report

**DOI:** 10.1186/1752-1947-8-68

**Published:** 2014-02-25

**Authors:** Yu-Hsuan Lin, Kuo-Ping Chang

**Affiliations:** 1 Department of Otolaryngology, Head and Neck Surgery, Kaohsiung Veterans General Hospital, 386 No., Ta-Chung 1st Rd., Kaohsiung 813, Taiwan

**Keywords:** Endomyocardial biopsy, Foreign body, Prevertebral space, X-ray image intensifier

## Abstract

**Introduction:**

Although inspirated or ingested foreign bodies constitute a common otolaryngologic emergency, the removal of a solitary retained foreign body from the neck has seldom been described in the literature. The ingestion of foreign bodies commonly results in perforated viscose or extraluminal migration to adjacent structures quite a long period of time after the fact. To the best of our knowledge, this is the first English language description of an endomyocardial biopsy complicated by a retained foreign body deep in the prevertebral space of the patient’s neck. We report such a case and share our experience in treating it.

**Case presentation:**

A 68-year-old Asian man suffering right-sided heart failure underwent an endomyocardial biopsy via his right internal jugular vein. After undergoing the procedure, he was found to have retained a metallic cup tip which had become lodged in his neck. A surgeon then performed neck exploration and the foreign body was removed without adverse effect.

**Conclusions:**

Decision making as to whether to remove the foreign body or not remains controversial. However, the later incidence of adhesive fibrosis or, even worse, of a catastrophic abscess or adjacent vascular injury might occur if the foreign body was not removed. Early exploration is suggested, if the patient’s condition makes this feasible.

## Introduction

Foreign bodies infrequently become lodged in the prevertebral space [1]. However, when this does occur, the presence of trauma or inflammation near the space makes its exploration dangerous because of the space’s anatomical proximity to the origin of the vertebral artery, the thyrocervical trunk, and the cervical sympathetic trunk and ganglion. In this case, we encountered rapid migration of a broken metallic cup tip into the prevertebral musculature after an endomyocardial biopsy (EMB); a type of case that has never been reported before. We also relied on the tremendously useful mobile C-arm (X-ray image intensifier) for intra-operative evaluation, to ensure the body’s safe surgical removal. The clinical history, radiological and intra-operative findings are presented.

## Case presentation

A 68-year-old Asian man presented to our cardiologic clinic with chief complaints of long-term swelling of his ankles, intermittent palpitations, and a propensity for becoming easily fatigued. On arrival, he presented the following vital signs: blood pressure of 121/71mmHg, body temperature of 35.9°C, and a heart rate of 52/minute. A physical examination revealed a grade III/VI blowing pansystolic murmur**,** best heard low over the left lower sternal border, an irregular heartbeat, jugular vein engorgement, and bilateral grade III pitting edema of his lower legs. The electrocardiogram showed sinus rhythm with frequent premature ventricular contractions. Color flow Doppler echocardiography showed an enlarged, hypokinetic right ventricle with a paper-thin right ventricular free wall and dilatation of his tricuspid valve annulus compatible with tricuspid regurgitation. Given the evidence from a serial laboratory study and image examination, the cardiologist had a high level of suspicion for arrhythmogenic right ventricular dysplasia. An EMB was arranged subsequently for sampling of the patient’s right ventricular pathological tissue via his right internal jugular vein. Using Seldinger’s technique, a 9 French silicone-locked catheter introducer sheet was applied. The bioptome was introduced via the sheet and then directed toward his right atrium. However, the cardiologist felt kinking while introducing the bioptome within the sheet just above the level of the patient’s right subclavian vein. A metallic cup tip was lost when the bioptome was withdrawn. The silicone introducer sheet was later removed without incident.

**Figure 1:**
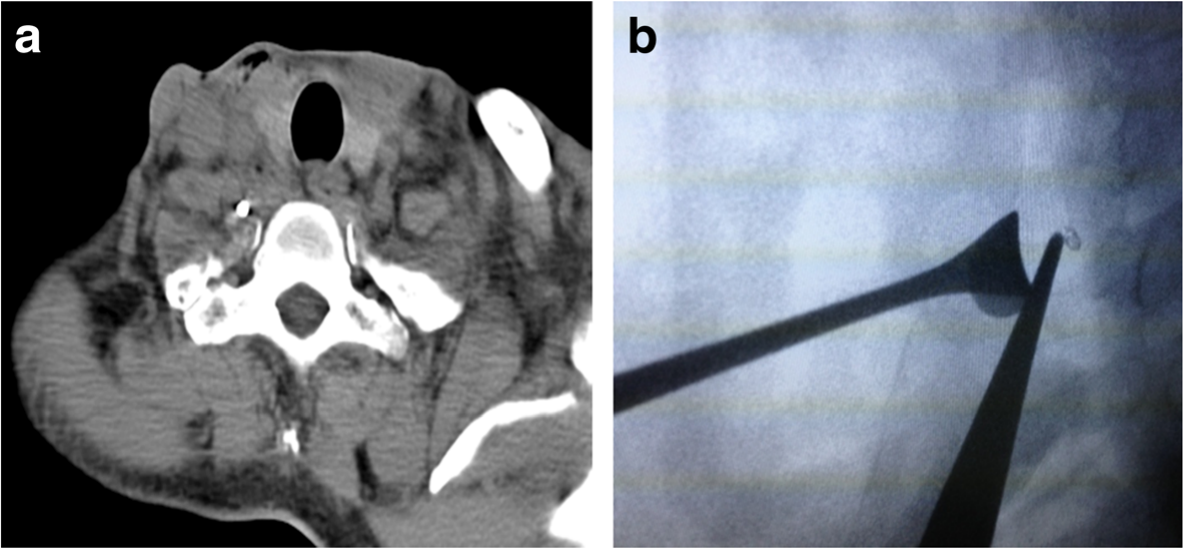
**Images of the foreign body. (a)** Plain computed tomography without contrast revealed a high density lesion impacted in the prevertebral space and its relationship with the carotid sheath and transverse process of the vertebral body. **(b)** X-ray image intensifier showed a radiopaque foreign body medial to the tip of the smooth pick-up, lateral to intervertebral discs of C7 and C8. A vein retractor holds the internal jugular vein posteriorly.

Out of concern for the floating foreign body inside the vasculature, an angiography via a right femoral approach was performed promptly, but no visible radiopaque foreign body was found within the patient’s vasculature with fluoroscopic guidance. He did not experience any immediate neurological or cardiovascular catastrophe. The cardiologist immediately arranged a computed tomography (CT) scan, and detected a high density lesion (Figure 1a) within the patient’s prevertebral musculature.

**Figure 2:**
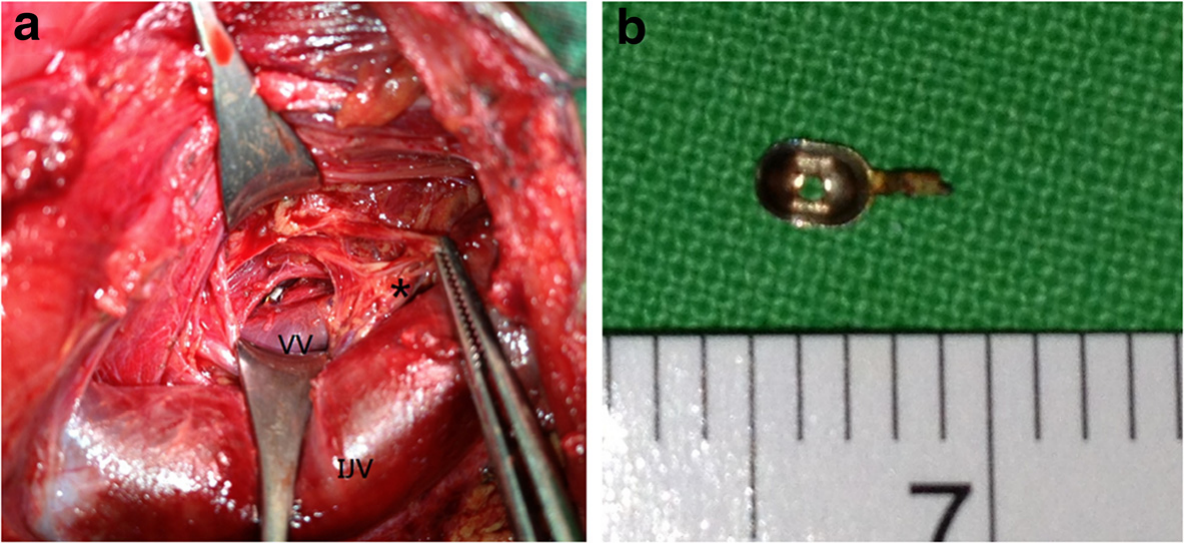
**Intraoperative findings. (a)** Intraoperative imaging showed the metallic cup tip adjacent to the vertebral vein buried within the prevertebral musculature; note the anatomic relationship of the sympathetic trunk (*) and the internal jugular vein. **(b)** Photography of the extracted specimen showed a 0.4 × 0.2 × 0.1cm metallic cup. Abbreviations: IJV, internal jugular vein; VV, vertebral vein.

The head-and-neck surgeons arranged neck exploration the next day. Out of consideration for safe surgical removal, we adopted a right lateral cervical approach. After developing superior and inferior subplatysmal flaps, the patient’s carotid sheath was opened by retracting the sternocleidomastoid muscle posteriorly. We explored between his internal jugular vein and his common carotid artery after identifying the vagus nerve, and then continued dissection deep down to the buccopharyngeal fascia. However, we were unable to approach the foreign body using physical digital examination, even after identifying the vertebral transverse process and sympathetic trunk. We then applied a mobile C-arm (X-ray image intensifier, Figure 1b) to check the exact three-dimensional relationship of the foreign body to the vertebral body. We then identified the missing metallic cup tip, just medial to a vertebral vein**,** and stuck within the deep prevertebral muscles (Figure 2a). The patient underwent surgery to remove the metallic cup tip (Figure 2b) uneventfully, with no immediate sequela. He was discharged 10 days later. The wound healed well during follow-up.

## Discussion

EMB is widely used for surveillance of cardiac allograft rejection and for the diagnosis of unexplained ventricular dysfunction. Typically, EMB is performed through the jugular or femoral veins, and is associated with a serious acute complication rate of less than 6% in most case series. Reported complications include perforation, pericardial tamponade, tricuspid valve damage, bleeding at the puncture site, pneumothorax, arrhythmias and coronary artery to right ventricle fistulae [2]. However, no retained foreign body during an EMB has been reported so far. The prevertebral plane is the deepest limit of our dissection. It is usually exposed in demolition surgery on the pharynx or in the drainage of retropharyngeal lymph node stations. The most important anatomical structures are the cervical sympathetic trunks and the vertebral artery. Direct trauma might lead to Claude Bernard-Horner’s syndrome and vertebrobasilar insufficiency. Because of the special anatomy of the prevertebral space, a surgical approach to this region requires careful consideration.

After reviewing the previously published English literature, we are able to contribute to the understanding of the etiologies of foreign bodies lodged in the neck as a result of ingestion, trauma and iatrogenesis. The mechanisms include extraluminal migration [1,3-5] and direct penetration [6]. In this case, we believe the presence of the foreign body should be attributed to rapid extraluminal migration to the adjacent structure. There is no consensus when it comes to the decision as to whether to remove the foreign body or not. However, removal at a later stage is hazardous, owing to adhesion of normal anatomic planes by fibrous tissue. In addition, delayed abscess formation [1,3,4], adjacent vascular aneurysm formation [5,7] and even malignant transformation process [8] have been reported in conjunction with prolonged foreign body retention. The evidence from these reported cases supports early surgical removal whenever possible. The choice of surgical approaches, inclusive of endoscopy, and an external approach**,** depends on the sizes and locations of the foreign bodies. Here, we used a lateral neck incision made directly to the carotid sheath to minimize unnecessary damage.

Preoperative assessment is important, and conventional radiography is capable of quite accurately defining the location of metallic foreign bodies. Three-dimensional [9] CT, and particularly three-dimensional enhanced CT virtual anatomy imaging [10], has been reported as having incrementally greater value in further diagnosis. Even so, actual exploration may be difficult to carry out when unpredictable conditions are encountered.

## Conclusions

To sum up, an EMB is an invasive but safe and trustworthy diagnostic procedure. It represents the gold standard for providing a definitive diagnosis in cases of disease entities like myocarditis, in cardiac allograft rejection, and in infiltration/storage myocardial disorders. Iatrogenic complication is seldom encountered [2]. Here, we present the first reported case describing an EMB with a retained foreign body in the neck. This case is not only informative both for the cardiologist performing EMB, but also highlights the importance of imaging for head-and-neck surgeons considering extracting a metallic foreign body deep in the prevertebral space. Precise preoperative CT evaluation and intra-operative guidance can assist with a foreign body’s safe surgical removal.

## Consent

Written informed consent was obtained from the patient for publication of this case report and any accompanying images. A copy of the written consent is available for review by the Editor-in-Chief of this journal.

## Abbreviations

CT: Computed tomography; EMB: Endomyocardial biopsy.

## Competing interests

The authors declare that they have no competing interests.

## Authors’ contributions

The patient was under the care of CKP. LYH and CKP surgically operated on the patient. LYH analyzed the data. LYH wrote the manuscript. LYH made additions to the manuscript. All authors reviewed and approved the final manuscript.

## References

[B1] Jiannis H Panagiotis K Vassiliki F Stavrianou E Iatrogenic migration of an impacted pharyngeal foreign body of the hypopharynx to the prevertebral space Int J Otolaryngol 2011 2011 274102 22187562 10.1155/2011/274102 PMC3236489

[B2] Aaron MF Joseph JM Charanjit SR Current status of endomyocardial biopsy Mayo Clin Proc 2011 86 1095 1102 10.4065/mcp.2011.0296 22033254 PMC3203000

[B3] Chen CY Peng JP Esophageal fish bone migration induced thyroid abscess: case report and review of the literature Am J Otolayrngol 2011 32 253 255 10.1016/j.amjoto.2010.02.006 20434801

[B4] Chung SM Kim HS Park EH Migrating pharyngeal foreign bodies: a series of four cases of saw-toothed fish bones Eur Arch Otorhinolaryngol 2008 265 1125 1129 10.1007/s00405-007-0572-x 18175137

[B5] Mathur NN Joshi RR Nepal A Rauniyar RK Common carotid artery pseudoaneurysm formation following foreign body ingestion J Laryngol Otol 2010 124 684 686 10.1017/S0022215109992283 20003600

[B6] Enomoto K Nishimura H Inohara H Murata J Horii A Doi K Kubo T A rare case of glass foreign body in the parapharyngeal space: pre-operative assessment by contrast-enhanced CT and three-dimensional CT images Dentomaxillofac Radiol 2009 38 112 115 10.1259/dmfr/69946733 19176654

[B7] Wang S Liu J Chen Y Yang X Xie D Li S Diagnosis and treatment of nine cases with carotid artery rupture due to hypopharyngeal and cervical esophageal foreign body ingestion Eur Arch Otorhinolaryngol 2013 270 1125 1130 10.1007/s00405-012-2138-9 22886383

[B8] Kalinich JF Emond CA Dalton TK Mog SR Coleman GD Kordell JE Miller AC McClain DE Embedded weapons-grade tungsten alloy shrapnel rapidly induces metastatic high-grade rhabdomyosarcomas in F344 rats Environ Health Perspect 2005 113 729 734 10.1289/ehp.7791 15929896 PMC1257598

[B9] Tao K Xu S Liu XY Liang JL Qiu T Tan JN Che JH Wang ZH Small metal soft tissue foreign body extraction by using 3D CT guidance: a reliable method Eur J Radiol 2012 81 3339 3343 10.1016/j.ejrad.2012.01.002 22321905

[B10] Yang XJ Xing GF Shi CW Li W Value of 3-dimensional CT virtual anatomy imaging in complex foreign body retrieval from soft tissues Korean J Radiol 2013 14 269 277 10.3348/kjr.2013.14.2.269 23483807 PMC3590339

